# A microfluidic device for simultaneous detection of enzyme secretion and elongation of a single hypha

**DOI:** 10.3389/fmicb.2023.1125760

**Published:** 2023-03-03

**Authors:** Ayaka Itani, Yosuke Shida, Wataru Ogasawara

**Affiliations:** ^1^Department of Bioengineering, Nagaoka University of Technology, Nagaoka, Japan; ^2^Department of Science of Technology Innovation, Nagaoka University of Technology, Nagaoka, Japan

**Keywords:** filamentous fungi, hyphal growth, cellulase, microfluidic device, real-time monitoring

## Abstract

Filamentous fungi grow through elongation of their apical region by exocytosis and secrete enzymes that can be of commercial or industrial importance. Their hyphae exhibit extensive branching, making it difficult to control hyphal growth for observation and analysis. Therefore, although hyphal morphology and productivity are closely related, the relationship between the two has not yet been clarified. Conventional morphology and productivity studies have only compared the results of macro imaging of fungal pellets cultured in bulk with the averaged products in the culture medium. Filamentous fungi are multicellular and their expression differs between different hyphae. To truly understand the relationship between morphology and productivity, it is necessary to compare the morphology and productivity of individual hyphae. To achieve this, we developed a microfluidic system that confines hyphae to individual channels for observation and investigated the relationship between their growth, morphology, and enzyme productivity. Furthermore, using *Trichoderma reesei*, a potent cellulase-producing fungus, as a model, we developed a cellulase detection assay with 4-MUC substrate to detect hyphal growth and enzyme secretion in a microfluidic device in real time. Using a strain that expresses cellobiohydrolase I (CBH I) fused with AcGFP1, we compared fluorescence from the detection assay with GFP fluorescence intensity, which showed a strong correlation between the two. These results indicate that extracellular enzymes can be easily detected in the microfluidic device in real time because the production of cellulase is synchronized in *T*. *reesei*. This microfluidic system enables real-time visualization of the dynamics of hypha and enzymes during carbon source exchange and the quantitative dynamics of gene expression. This technology can be applied to many biosystems from bioenergy production to human health.

## 1. Introduction

Filamentous fungi are used as microbial cell factories to produce antibiotics, organic acids, and enzymes (Meyer et al., 2016). Many of these products constitute a multi-billion-dollar industry, the value of which is expected to increase with the transition from petroleum to a bio-based global economy (Cairns et al., 2018). Therefore, increasing the productivity of their protein secretions is of tremendous industrial importance.

The growth of these fungi depends on the exocytosis of hyphal tips and their morphogenesis and secretion are linked (Bartnicki-Garcia and Lippman, 1969). Therefore, increasing the number of hyphal tips has been thought to help improve protein secretion (Te Biesebeke et al., 2005; He et al., 2016). However, increasing the number of hyphal tips does not necessarily correlate with increasing protein titer (Hayakawa et al., 2011; Read, 2011; Burggraaf et al., 2016). These filamentous fungi are multicellular organisms that are known to exhibit cellular heterogeneity (Avery, 2006; Munsky et al., 2012; Carson et al., 2013; Symmons and Raj, 2016) which may result from differences in cell development and cell cycle state, differences in cell age, and even from non-genetic factors such as nutritional status. Cellular heterogeneity has been shown to have a dramatic impact on fungal productivity (Naranjo-Ortiz and Gabaldón, 2020), but during routine measurements its influence on productivity is masked by the average response from a given population as studies on filamentous fungi are usually conducted on fungal aggregates cultured in flasks or jar fermenters using culture medium (Grimm et al., 2005; Veiter et al., 2018; Meyer et al., 2021). One approach to solving this issue is to analyze populations at the individual cell level.

Several methods have been attempted to address this issue. Some methods include randomly placing hyphae on glass substrates or agar blocks and observing them under a microscope (Roca et al., 2010; De Bekker et al., 2011; Bleichrodt et al., 2012). However, these methods lack temporal and spatial control over hyphal tracking because the hyphae are extensively branched and cannot be cultured for long periods. To quantify the dynamics of large cell populations and to understand cell-to-cell variability, new approaches that can confine large numbers of hyphae to individual channels and control their microenvironment are needed.

Microfluidics is an excellent tool for manipulating and analyzing single cells as it can confine cells within microstructures (Zare and Kim, 2010; Lecault et al., 2012; Bedekovic and Brand, 2021). Several microfluidic devices have been reported for observing single mycelia. Although these devices are suitable for observing the mycelium itself and its behavior (such as branching) within the fungus, it is difficult to observe their secretions due to limitations afforded by their structure (Held et al., 2010; Geng et al., 2015; Held et al., 2019; Baranger et al., 2020; Hopke et al., 2021). Droplets can be used for observing the secretions of filamentous fungi, but they confine a single spore, making it impossible to observe the secretions of a single mycelium. In addition, as the mycelium grows, it breaks through the soft wall of the droplet, making it impossible to incubate for extended periods (Beneyton et al., 2016; He et al., 2019).

To overcome these problems, we have developed a microfluidic platform that can observe hyphal growth and secretion dynamics simultaneously. It combines a device with spatially separated flow paths to capture single hypha and cellulase detection in a microspace using 4-methylumbelliferyl β-d-cellobioside (4-MUC). With this method, we detected the secretions of cells in microfluidic channels, which had been considered a challenge so far.

Further, this study aimed to develop a platform to elucidate the relationship between the growth and morphology of the hypha itself and its enzyme productivity. By culturing a model filamentous fungus *Trichoderma reesei* (teleomorph *Hypocrea jecorina*, Ascomycota) that secretes large amounts of cellulolytic enzymes on our advanced microfluidic platform, we have shown the growth/cellulase expression dynamics over time in response to carbon source switching for the first time. Elucidating the behavior and molecular regulation of filamentous hypha is critical for various applications in biotechnology and bioenergy production. The present results provide a new basis for elucidating the hyphal and exocrine behavior of *T*. *reesei*.

## 2. Materials and methods

### 2.1. Fungal strains and growth conditions

*Trichoderma. reesei* strains QM9414 (ATCC26921) and PC-3-7 (ATCC66579) used in this study were obtained from Kao Co., Ltd. Previously described *pyr4* disruptants QM9414ΔP and PC-3-7ΔKP (Hirasawa et al., 2019) were used as the host for transformation. Strains were grown on potato dextrose agar (PDA) plates and harvested conidia. Conidia of *T*. *reesei* strains were inoculated into basic medium (1% appropriate carbon source, 0.14% (NH_4_)2SO_4_, 0.2% KH_2_PO_4_, 0.03% CaCl_2_•2H2O, 0.03% MgSO_4_•7H_2_O, 0.05% Bacto Yeast Extract, 0.1% Bacto Peptone, 0.1% (w/v) Tween 80, 0.1% (w/v), 50 mM tartrate buffer (pH4.0), and trace element). Trace element comprised 0.006% H_3_BO_3_, 0.026% (NH_4_)6Mo_7_O_24_•4H_2_O, 0.1%FeCl_3_•6H_2_O, 0.4% CuSO_4_•5H_2_O, 0.008% MnCl_2_•4H_2_O, 0.2% ZnCl_2_.

### 2.2. Biochemical analyses

To obtain the pattern of secreted protein, the culture supernatant was subjected to SDS-PAGE (Laemmli, 1970). The Precision Plus Dual Color Standard Marker (Bio-Rad) was used as molecular weight standard. Protein concentration was determined by Bradford method using bovine gamma globulin as the standard (Bradford, 1976).

### 2.3. Construction of plasmids and strains

For the GFP-H2B expression, the gene encoding H2B (*h2b*) and its 2 kbp upstream and downstream regions were amplified by PCR with the genomic DNA derived from QM9414 as the template. This fragment was inserted into the HinCII site of the pUC118 vector by the Gibson Assembly system (New England Biolabs) to create pU*h2b*. An inverse PCR was then performed for the linearization of pU*h2b* at the start codon of *h2b* and the gene encoding AcGFP1 was introduced into just upstream of *h2b*. The resulting plasmid, pU*h2b-gfp*, was subjected to an inverse PCR to open the downstream region of *h2b*, and the selection marker gene *pyr4* was inserted to obtain plasmid pU*h2b-gfp-pyr4*. The GFP-H2B expression cassette was released from pU*h2b-gfp-pyr4* by XbaI and SbfI. This expression cassette was used to transform the QM9414ΔP strain.

For the CBHI-GFP expression, the gene encoding CBHI (*cbh1*) and its 2 kbp upstream and downstream regions were amplified by PCR. This fragment was inserted into the EcoRV site of the pBluescript II KS (+) vector by the Gibson Assembly system to create pB*cbh1*. An inverse PCR was then performed for the linearization of pB*cbh1* at 318 bp downstream of *cbh1* to ligate the selection marker gene *pyr4*. The resulting plasmid, pB*cbh1-pyr4*, was subjected to an inverse PCR to open at the site between *cbh1* and its terminator, and the gene encoding AcGFP1 was inserted to obtain the plasmid pB*cbh1-gfp-pyr4*. The CBH1-GFP expression cassette was released from pB*cbh1-gfp-pyr4* by ClaI and SpeI. Using this expression cassette, PC-3-7ΔKP strain was transformed and PC*cbh1gfp* strain was obtained. The primers used in this study are shown in Supplementary Table S1 and the plasmid map is shown in Supplementary Figure S1.

### 2.4. Fungal transformation

Transformation of *T*. *reesei* protoplasts was carried out by the protoplast-PEG method as previously described (Shida et al., 2015) with a small modification in which Yatalase (Takara bio) was used for cell wall digestion for preparing the protoplasts. Protoplasts were plated on minimal medium without uridine to screen for cells with uridine autotrophy. Candidates of transformants were streaked twice on minimal medium without uridine to obtain stable transformants. Recombination of strains was confirmed by Southern hybridization analysis using an AlkPhos Direct kit (Cytiva).

### 2.5. Microfluidic device fabrication

The microfluidic devices were fabricated by standard photolithographic techniques (details in Supplementary Figure S2; Friend and Yeo, 2010; Qin et al., 2010). The molds for the PDMS devices were fabricated in two steps using SU-83205 and 30,025 on silicon wafers. PDMS (SYLGARD 184 Silicone Elastomer Kit) was poured into the SU-8 mold in a container and allowed to cure. Holes were drilled and bonded together with coverslips (24× 60 mm, Matsumani) activated with a plasma cleaner (PDC-32G, Harrick Plasma).

### 2.6. Operation of the microfluidic device

*Trichoderma reesei* conidia were suspended in basic medium containing 1% glucose and left to swell at 28°C for about 5 h. The conidia obtained by centrifugation were then suspended in basic medium and injected into the microfluidic device. Then, the basic medium containing appropriate carbon source (1% glucose, CMC or cellobiose) and 1 mM 4-MUC was constantly perfused into the microfluidic device at a flow rate of 0.5 ml/h using a syringe pump (YSP-301, YMC).

### 2.7. Image acquisition and analysis

The microfluidic device was mounted on the stage of a Nikon Ti2-C2 confocal microscope equipped with 60× (Apo Lambda S Oil, Nikon) and 100× (Apo TIRF Oil, Nikon) objective lenses. GFP was excited by a 488 nm laser and detected at 525 nm; Calcofluor White (CFW) and 4-MU were excited by a 405 nm laser and detected at 438 nm. Crosstalk was minimized by the use of line sequential mode. The images were stored and manually analyzed using the Nikon NIS-Elements software, which is compatible with the microscope. Fluorescence intensity was calculated by averaging over a region of interest (ROI) of 80 pixels or more. In the measured wavelength range, the autofluorescence of the hypha and the culture medium was weak and negligible compared to the fluorochrome.

### 2.8. Characterization of molecular diffusion

The microfluidic device was first filled with basic medium, and then perfused with fluorescent staining reagents CFW solution into the medium infusion channel using a syringe pump. Fluorescence images of hyphal growth channels were captured after CFW introduction, and the intensity was quantified.

## 3. Results

### 3.1. Microfluidic device design

**Figure 1:**
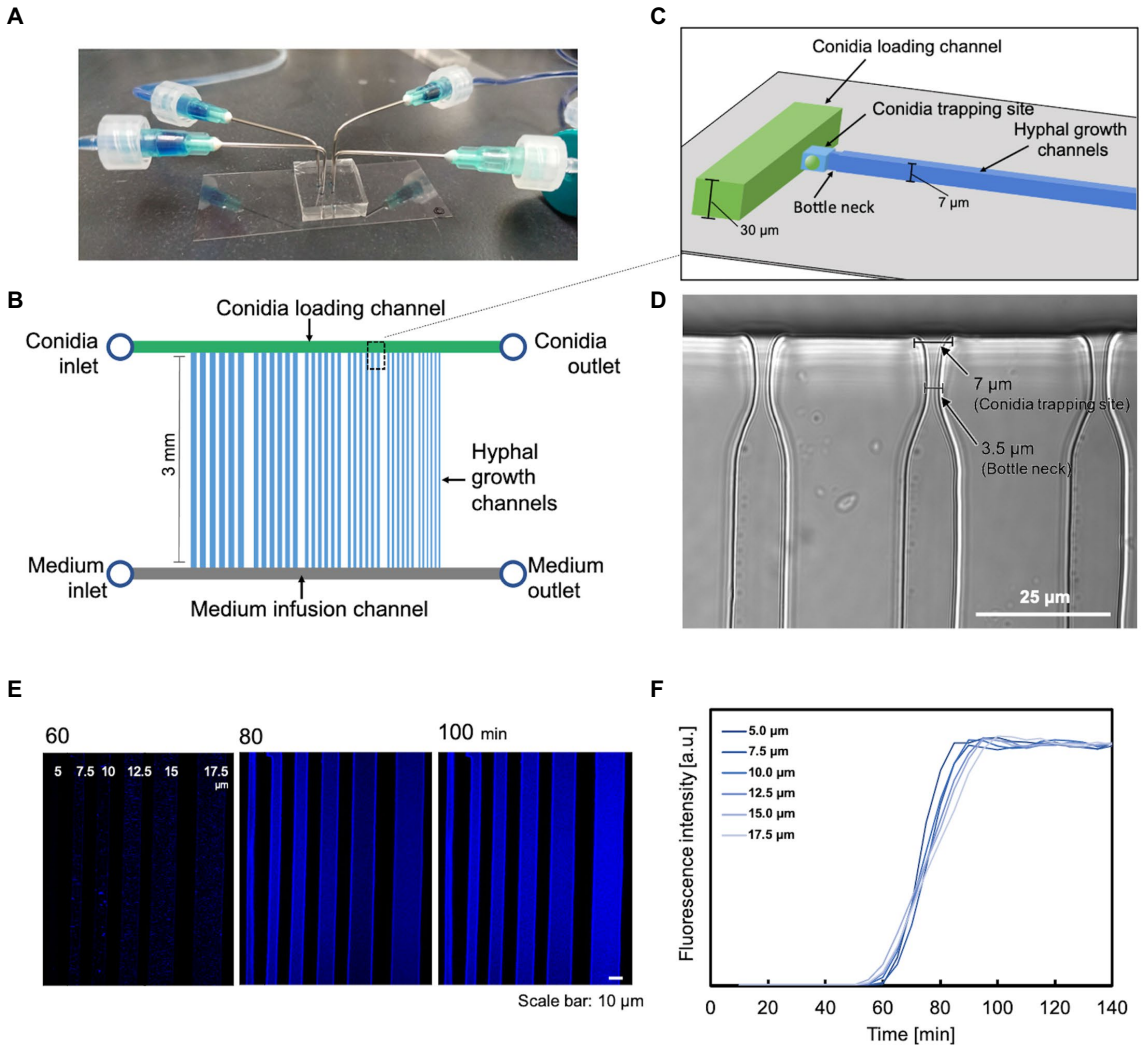
A microfluidic device for analyzing the growth and dynamics of a hypha. **(A)** External view of the microfluidic device. **(B)** Schematic representation of the chip layout: it consists of two inlets for supplying cells and medium, respectively, two outlets, a parallel array of conidia supply channels (green), medium injection channels (gray), and hypha growth channels (blue). **(C)** Schematic representation of the conidia trapping site containing two short channels of different dimensions (light blue) connecting the conidia supply channel (green) and the hypha growth channel (blue). The narrower and shallower channel traps a single conidium. **(D)** Image of the constricted structure for conidia trapping. Scale bar: 25 μm. **(E,F)** Molecular diffusion properties on microdevices with different channel widths. **(E)** Visualization of the gradient profile. Scale bar: 10 μm. **(F)** Quantification of the gradient profile of CFW diffusion to steady state. The measurements were carried out in the middle of the channel, just below the spore trap site.

We used multilayer soft lithography to fabricate a “conidia trap” microfluidic device (Figure 1A) composed of polydimethylsiloxane (PDMS), which allows for the observation of a single hypha in real-time. The device consists of three major regions. The first region is the conidia loading channel (width 200 μm × height 30 μm, Figures 1B,C, green) with a “conidia inlet” for the delivery of conidia and a “conidia outlet” to release the excess conidia. The second region is a channel with a “bottleneck” design for conidia capture (conidia trapping site: length 7.0 μm × width 7.0 μm, bottleneck: length 5.0 μm × width 3.5 μm, Figures 1C,D, light blue), allowing the observation of a single hyphal elongation into the observation channel (length 3.0 mm × a series of width). The widths of the channels were set from 5.0 μm to 17.5 μm with increments of 2.5 μm. The hypha observation channel (Figure 1B, blue) had a height of 7.0 μm which was the maximum diameter of the hypha to keep the hypha in focus. The third area is a channel (200 μm wide × 30 μm high, Figure 1D, gray) with a “medium inlet” for the introduction of medium and a “medium outlet” to release the medium and waste.

Our system also provided a stable and constant environment during the experiment, facilitating the observation of hypha. Molecular diffusion was measured with this device. When CFW was injected from the medium inlet at a flow rate of 0.5 ml/h, diffusion was completed at approximately the same time for all channels (Figures 1E,F). The slightly faster rate in the narrower channels is due to the hydrodynamic properties: the closer the growth channel is to the medium inlet, the more rapid molecular diffusion starts, and the thinner the growth channel, the faster molecular diffusion is completed.

### 3.2. Characterization of microfluidic devices

**Figure 2:**
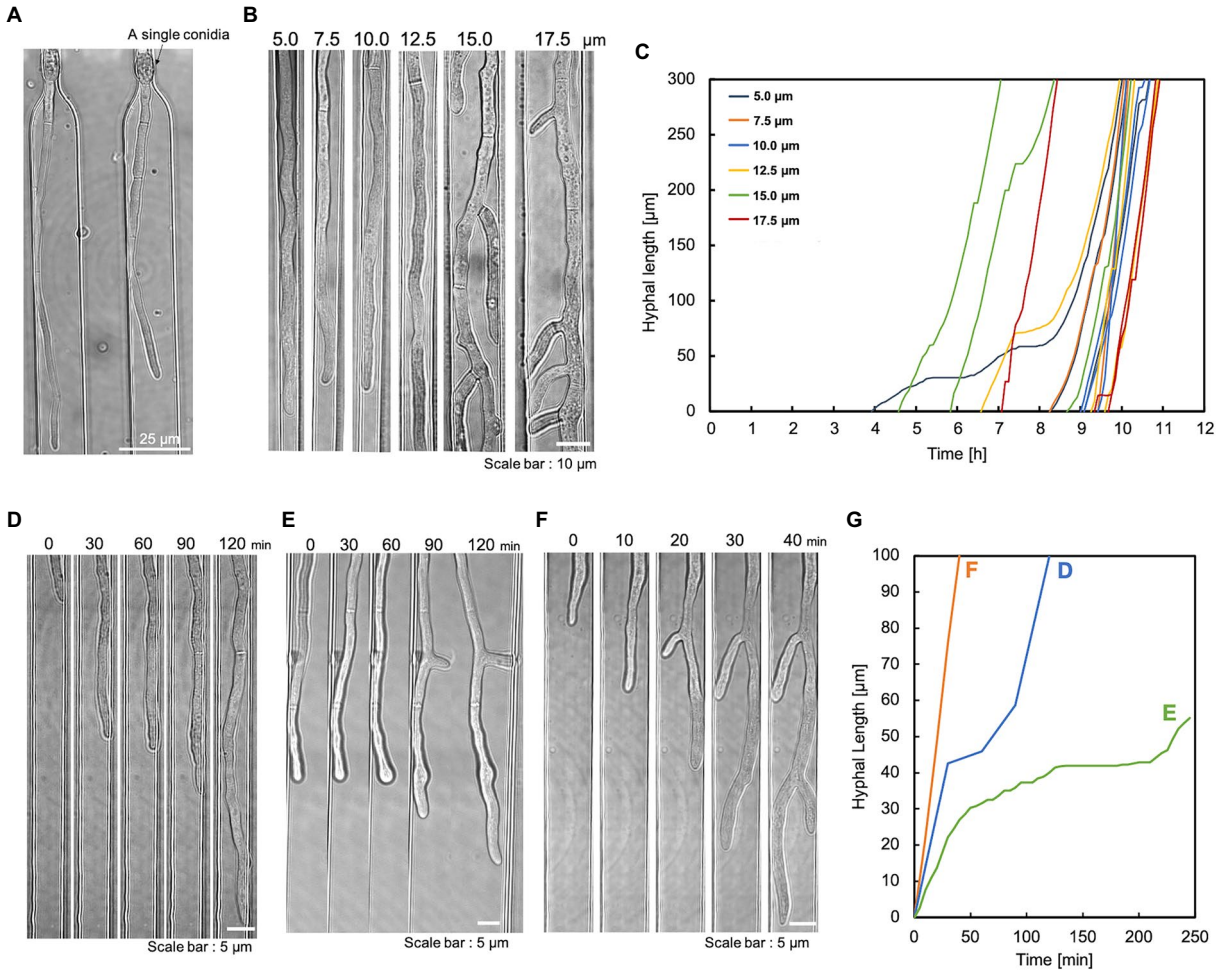
Single conidia trapping and compartmentalized hyphal growth. The hyphae were cultivated under a constant flow of 1% glucose in the basic medium at 0.5 mL/h. **(A)** A single conidium of *T*. *reesei* was trapped at the conidia trapping site. Scale bar: 25 μm. **(B)** The compartmentalized hyphal extension of *T*. *reesei* along the hyphal growth channels. Observation at 11 h. Scale bar: 10 μm. **(C)** Time course of three individual hyphal length elongations per channel width on a single device. The hyphal length was measured at regular intervals of 5 min starting after conidia trapping. **(D)** Delayed elongation without branching. Scale bar: 5 μm. **(E)** Delayed elongation with branching. Scale bar: 5 μm. **(F)** Hypha that did not delay elongation with branching. Scale bar: 5 μm. **(G)** Quantification of hyphal elongation in **(D–F)**.

The apical tip of the hypha is the elongation point and the site of enzyme secretion. Therefore, this device was designed to trap conidia at the hyphal growth channel’s entrance and observe the hyphal elongation. The conidia of *T*. *reesei* have an initial diameter of about 3 μm and they swell to about 10 μm before germination. Therefore, the conidia were inoculated into the basic medium containing 1% glucose, left at room temperature for 5–6 h to swell to about 5 μm, and washed in the basic medium without the carbon source. The swollen conidia were then suspended in the basic medium and introduced into the conidia inlet using a syringe. The slow-swelling or dead conidia slipped through the bottleneck structure and were ejected from the medium outlet. Consequently, most of the trapped conidia germinated. The germination time of conidia varied greatly, but the selective trapping made it possible to match the germination time to some extent (Figure 2B). Also, the bottleneck did not cause abnormal hyphal morphology (Figure 2A).

After the conidia loading channel was filled with conidia suspension, the conidia outlet was closed and pressurized with a syringe. In this way, a single conidium was retained in the conidia trap structure (Figure 2A). Once the trapping site was occupied, subsequent conidia could preferentially travel to the following empty trap structure. This method could achieve a trapping efficiency of over 70%. To induce the direction of conidial germination and hyphal growth into the medium infusion channel, the conidia inlet and outlet were closed after conidia trapping, and the medium was constantly perfused into the medium infusion channel. This compartmentalization eliminates cross-contamination due to cell–cell interactions and allows for independent and accurate analysis of individual hypha.

We evaluated the hyphal extension of *T*. *reesei* QM9414 in the hyphal growth channels. The channel geometry had a significant impact on hyphal growth and morphogenesis. To assess the effect of the confinement level, we cultivated *T*. *reesei* in a device with varying widths (5.0, 7.5, 10.0, 12.5, 15.0, and 17.5 μm). The hyphae growing in wider channels (15–20 μm) generated significant branching, which interfered with the visualization of the leading hyphae (Figure 2B). Hyphae growing in 5 μm channels occupied the entire channel width, obscuring their morphology (mainly cell boundaries). The length of individual hypha growing in each channel was measured as a function of time. The hyphal elongation rates at different channel widths were comparable. Still, there were occasional delays in elongation (Figure 2C). Hyphae were observed during the elongation delay [without branching (Figure 2D) and during branching (Figure 2E)]. In addition, the delay did not necessarily occur during branching (Figures 2F,G).

Furthermore, this system was also helpful for exploring intracellular processes (Supplementary Figure S3). Real-time observation of nuclear organization and migration using *T*. *reesei* QM9414 with the histone H2B-GFP nuclear marker showed that the system could be used for prolonged observation (for >20 h after switching media and > 30 h from the start of incubation) without imposing nutrient depletion or shear stress on the hypha and with minimal displacement due to media flow.

### 3.3. Investigation of detection methods for cellulases

**Figure 3:**
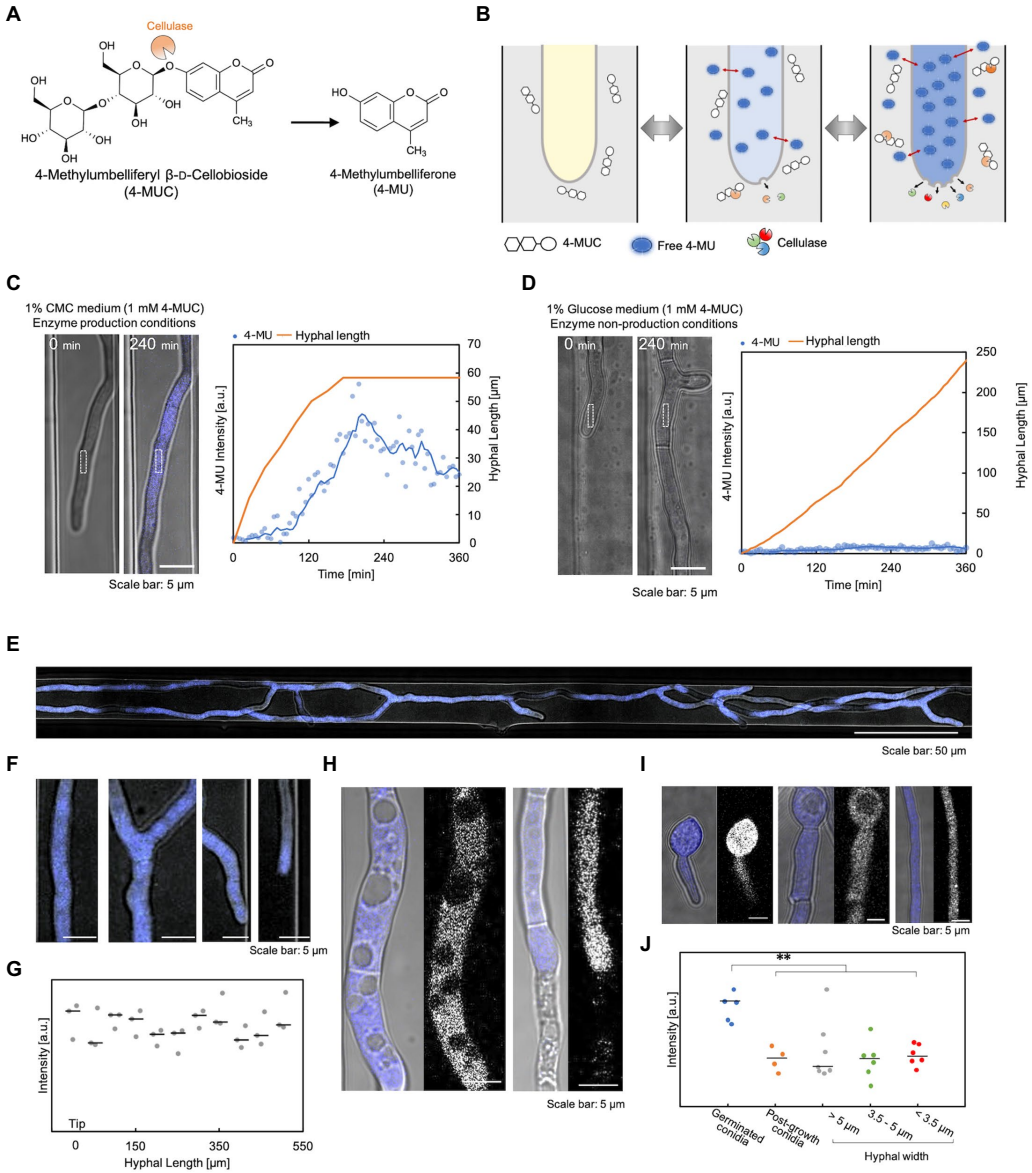
Verification of cellulase detection using 4-MUC. **(A)** 4-MUC releases 4-MU by cellulase. **(B)** Schematic illustration of the principle of cellulase detection by 4-MUC in the flow channel. **(C,D)** Hyphal growth and 4-MU intensity under the enzyme production condition **(C)** and under the enzyme non-production condition **(D)**. Left (microscopic image) and right (quantification of hyphal length and fluorescence intensity). **(E–J)** Differences in 4-MU fluorescence intensity in various hyphal conditions. **(E)** The overall image of the hypha. **(F)** Enlarged image of **(E)**. From left to right: hyphal base, branching, branched tip, and leading hyphal tip. **(G)** Fluorescence intensity from the tip (0 μm) to 500 μm. **(H)** 4-MU was not detected in vacuoles or dead cells. **(I)** Hyphal thickness and fluorescence intensity. From left to right: spores immediately after germination, spores and thick hyphae (hyphal width: about 5 μm) after hyphal elongation, and thin hyphae (hyphal width: about 3.5 μm). **(J)** Fluorescence intensity in hyphae of various widths. **p ≤ 0.01.

To investigate the correlation between hyphal growth and enzyme secretion, we devised a method that detected enzymes inside the device using 4-MUC. 4-MUC is cleaved by cellulase to release 4-methylumbelliferone (4-MU), which fluoresces and can be used to measure and screen cellulase activity (Figure 3A). 4-MU is a coumarin-based fluorescent substance, which is very hydrophobic and is hardly soluble in water. Therefore, it is necessary to use methanol or DMSO to measure the fluorescence intensity. When 4-MU and the hyphae were added to a 5% methanol solution, the solution fluoresced, but the hypha did not. However, when they were added to a medium free of organic solvents, the medium did not fluoresce, and strong fluorescence was observed in the hypha. In addition, the fluorescence intensity in the cytoplasm changed according to its concentration equilibrium (Figure 3B). The details of the cellulase detection assay using 4-MUC are shown in Supplementary Figure S4.

To confirm that 4-MU released by cellulase can be detected, we compared the fluorescence intensity of 1% glucose medium with 1 mM 4-MUC (cellulase non-production condition) and 1% carboxymethylcellulose (CMC) medium with 4-MUC (cellulase production condition). Under cellulase production conditions, the hyphal fluorescence intensity increased with time, and decreased when hyphal elongation ceased (Figure 3C). Under cellulase non-production conditions, the fluorescence intensity of the cytoplasm did not increase at all, even after hyphal elongation (Figure 3D). This observation demonstrated that the detection of cellulase by 4-MUC in the microdevice was possible. 4-MUC has no effect on growth, morphology, or enzyme productivity, as shown in Supplementary Figure S5.

To examine whether the fluorescence intensity of 4-MU is dependent on the hyphal condition, cultures were grown in 1% CMC medium containing 1 mM 4-MUC. After about 10 h of incubation, microscopic observation revealed uniform fluorescence throughout the hypha (Figures 3E,F). Fluorescence was measured every 50 μm from the tip of the hypha, and no significant difference in fluorescence intensity was observed (Figure 3G). No fluorescence was observed from vacuoles and dead hypha (Figure 3H). Fluorescence intensity was not affected by hyphal thickness. However, strong fluorescence was observed in the hypha immediately after germination (Figures 3I,J).

### 3.4. Tracking of cellulase dynamics inside and outside the hypha

Finally, we attempted monitoring of enzyme production and hyphal behavior. To study the relationship between the dynamics of enzyme production and hyphal morphology, it was necessary to increase the sensitivity of detection of morphological changes, expression of genes encoding enzymes, and secreted enzymes. Our results have shown that our device can analyze morphological variations, but we also encountered a few limitations. *T*. *reesei* shows the best performance for enzyme production when solid cellulose is used as a carbon source (Dashtban et al., 2011). In contrast, only soluble sugars can be used as culture substrates because of clogging in devices with small channels. Therefore, we decided to use the PC-3-7 strain, which has acquired high cellulase inductivity against not only microcrystalline cellulose and sophorose but also cellobiose (Nogawa et al., 2001). We monitored the cellulase expression behavior in this strain by fusing GFP to the C-terminus of cellobiohydrolase I (CBHI), the most abundant cellulase produced.

**Figure 4:**
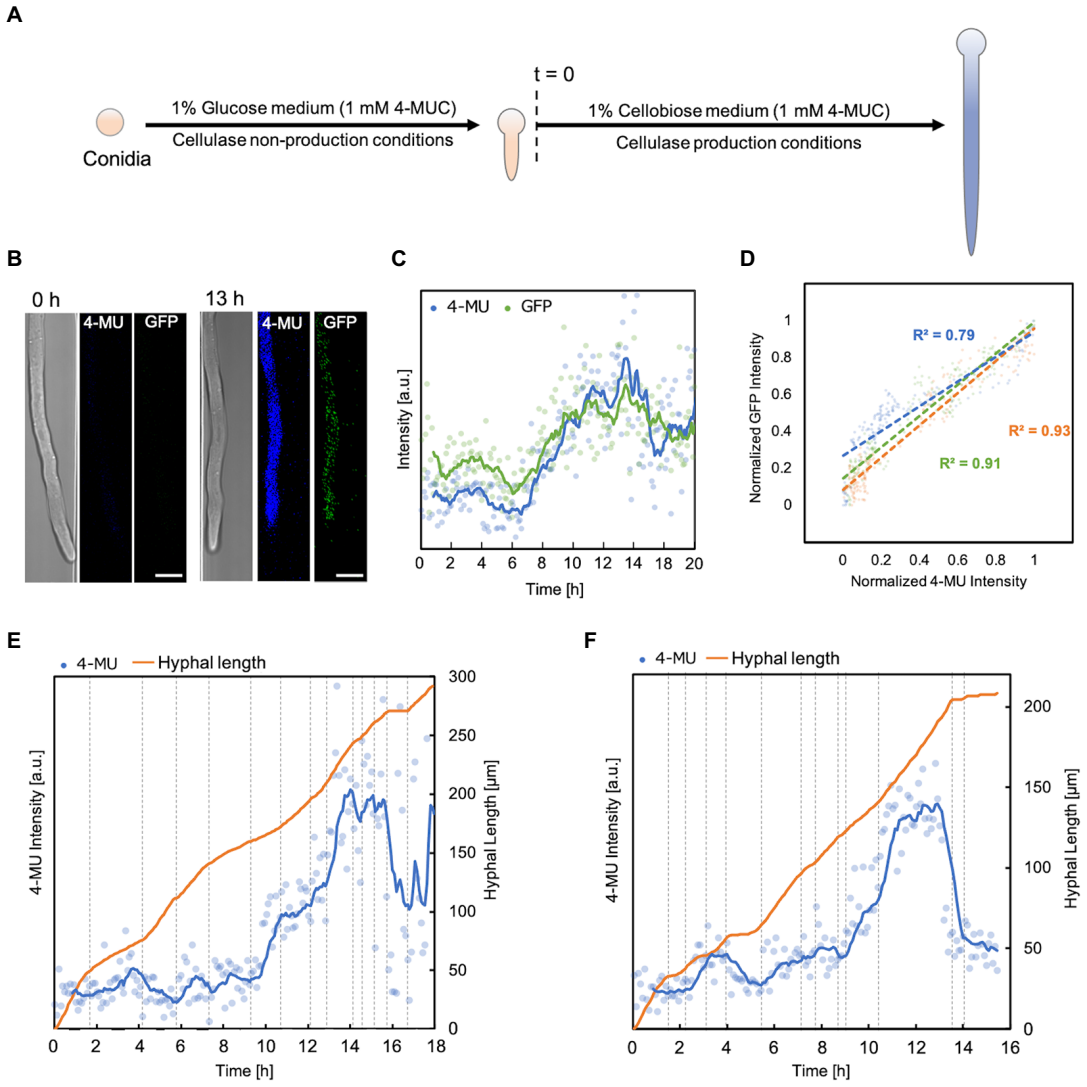
Correlation between fluorescence intensity and elongation of hypha. **(A)** Flowchart of enzyme production and hyphal elongation measurements in the 4-MUC assay. Time of switch to enzyme production medium (*t* = 0). **(B)** Fluorescence images from 4-MUC assay using PC*cbh1gfp* strain at the time of medium switch (*t* = 0 h) and at the time when the highest fluorescence was observed (*t* = 13 h). Scale bar: 5 μm. **(C)** Fluorescence intensity of 4-MU and GFP in hypha. Channel width: 7.5 μm. **(D)** The normalized plot of 4-MU and GFP fluorescence intensity. Channel width: 15 μm (blue), 10 μm (green), and 7.5 μm (orange). **(E,F)** 4-MU fluorescence intensity and hyphal elongation profile. Channel width: 7.5 μm **(E)**, 15 μm **(F)**.

Experiments were carried out steadily for about 40 h. To ensure that each hypha produced the enzyme simultaneously, the first incubation period was carried out in a non-enzyme-producing medium (1% glucose, 1 mM 4-MUC). When the hyphal length reached about 150 μm, the medium was switched to an enzyme-producing medium (1% cellobiose, 1 mM 4-MUC; Figure 4A). Fluorescence intensity and hyphal growth were measured every 5 min. The maximum fluorescence intensity was reached about 13 h after switching the medium in all hyphae (Figures 4B,C). Normalized plots of GFP fluorescence intensity versus 4-MU fluorescence intensity showed a strong correlation (*R*^2^ = 0.7 ~ 0.9, Figure 4D). 4-MU fluorescence intensity began to increase from about 2 h (Figures 4E,F). According to the molecular diffusion profile of the device (Figure 1F), this was immediately after the medium exchange was completed. Subsequently, the intensity trend of 4-MU increased with increasing hyphal elongation rate and decreased with decreasing elongation rate or cessation of hyphal elongation. The fluorescence intensity of 4-MU did not match in each channel (Figures 4E,F). This indicated that each channel was compartmentalized and that the extracellular enzyme profile could be separately measured. The hypha continued to grow with some fluctuations, and no death or lysis was observed during the observation period.

## 4. Discussion

This study introduces a novel microfluidic device for the morphological observation and quantification of enzyme production in the filamentous fungus *T*. *reesei*. This microfluidic device offers unprecedented technical advantages for studying hyphal growth and dynamics. First, medium and other components are continuously supplied to the hypha by diffusion rather than directly, preventing shear stress and hyphal displacement (Figure 1F). In addition, by capturing one similarly sized conidium at a time, each hypha could be divided into separate compartments, allowing for independent and accurate measurements (Figure 2A). The layout of the microfluidic device, which combines these features, enables stable and robust long-term operation for semi-automatic, simultaneous monitoring of different hypha using a multipoint time-lapse. Previous microfluidic devices have specialized in observing mycelial behavior, but are unable to visualize secretions (Held et al., 2010; Geng et al., 2015; Held et al., 2019; Baranger et al., 2020; Hopke et al., 2021). This new system allows detailed, real-time observation and analysis of cell-to-cell variation and heterogeneity during hyphal growth, the distribution and dynamics of intracellular organelles, long-term protein expression dynamics, and cellular responses to well-defined environmental signals and stimuli.

This study aimed to develop a platform that allows the relationship between the growth of the target hypha and enzyme production. The detection method devised for monitoring enzyme production in this study relies on the difference in polarity between water and hypha. 4-MUC is used for detecting cellulases, which are degraded by cellobiohydrolase, endoglucanase, and beta-glucosidase. The degradation product 4-MU is almost insoluble in water, and it is common to dissolve 4-MUC in a lower polar solution such as methanol or DMSO for the enzymatic reaction (Garvey et al., 2014). 4-MU has been used in many screening methods and enzyme activity assays, but there have been no reports of 4-MU being localized in the cytoplasm. Therefore, when the enzymatic reaction releases 4-MU, it is localized in the solution, which is less polar than the cytoplasm. However, our experiments showed that the culture medium without organic solvents was highly polar. When 4-MU was added to a 5% methanol solution and hyphae were soaked, no fluorescence was detected in the hypha, but the solution emitted fluorescence. When 4-MU was added to a methanol-free solution and hyphae were immersed, cytoplasmic fluorescence increased immediately (Supplementary Figure S4A). Therefore, it is possible that a tiny amount of dissolved 4-MU entered the cell through the plasma membrane by passive transport. The topological polar surface area value (calculated by Molinspiration Cheminformatics, Slovensky Grob, Slovak Republic) was 208.74 Å^2^ for 4-MUC and 50.44 Å^2^ for 4-MU, indicating that 4-MU had higher membrane permeability. This indicates that the trace amounts of 4-MU degraded by cellulase are detectable, and the detection sensitivity is very high and immediate.

The hydroxy group of 4-MU facilitates the chemical synthesis of various fluorescently labeled substances. Typical examples are 4-methylumbelliferyl phosphate (4-MUP), 4-methylumbelliferyl-β-d-glucopyranoside (4-MUG), and 4-methylumbelliferyl acetate (MU-Ac). These are used for various purposes. An essential finding of this study is that in the absence of methanol or DMSO, 4-MU localizes to the cytoplasm and the fluorescence intensity in the cytoplasm changes according to its concentration equilibrium. This allows the detection of trace amounts without the adverse effects of organic solvents on hyphae. Hence the increased detection sensitivity means that reactions missed by previously used fluorescent substrates can now be detected immediately.

We investigated the relationship between hyphal growth and enzyme expression using the novel device and the method we developed to detect extracellular enzymes by 4-MUC. First, the fluorescence intensity of 4-MU did not match in each hypha, indicating that the extracellular enzyme profile of each hypha could be measured separately (Figures 4E,F). The set of cellulases of *T*. *reesei* is coordinately expressed, and the proportion of each expression amount does not change significantly (Foreman et al., 2003). Therefore, it is possible to confirm that other cellulases are also expressed by fusing GFP to one of the cellulases and measuring the fluorescence intensity. However, even if GFP-fused cellulase is secreted, the amount of secreted cellulase is so small relative to the amount of solution outside the hypha, and the change in fluorescence intensity in the solution not substantial enough, that the amount of enzyme is not accurately measured. Therefore, we created a PC*cbh1gfp* strain that expresses but does not secrete GFP-CBH1 to accurately determine the cellulase production of the hypha to be measured (Supplementary Figure S6). GFP that is not secreted is distributed in the cytoplasm but is not degraded intracellularly and eventually accumulates in the vacuole (Shoji et al., 2006, 2010). When we sampled flask cultures and observed them every 12 h, we found that GFP accumulated in the vacuole after 60 h. However, we could not find any large organelle with a specific increase in GFP fluorescence intensity before that time. Therefore, there should be no obstacle for observing hypha in the early stages of enzyme production in the microfluidic device. We did not observe any vacuoles, cessation of hyphal growth, or lysis during the 20-h observation period after switching the substrate of the medium flowing into the microfluidic device from glucose to cellobiose. It suggests that the GFP fluorescence intensity indicates the amount of CBHI produced *in situ* for a short period after the *cbh1* promoter was activated. 4-MU and GFP fluorescence intensity trends in the same hyphae showed a strong correlation (R^2^ = 0.7 ~ 0.9). Since the proportion of each cellulase does not vary significantly (Foreman et al., 2003), the amount of 4-MUC degraded by CBHI and secreted enzymes in the hypha could be reliably measured (Figures 4C,D). The growth of the fungus stopped or slowed down when the culture substrate or environment changed. Throughout the observation period, the fluorescence intensity of 4-MU increased with a faster hyphal growth rate and decreased with a slower growth rate. This indicates that exocytosis supply of cell wall components and secretory enzymes can be observed in real time.

However, there were cases in which the fluorescence intensity of 4-MU showed only a slight increase even when the hypha elongated at a high rate (Figures 4F, 5–7 h). Thus, the rate of hyphal elongation did not coincide with the rate of change of 4-MU fluorescence intensity, suggesting that the amount of enzyme in secretory vesicles is not constant. Further studies are needed to corroborate the inconsistent variation in hyphal elongation and enzyme production.

In this study, we showed that cellulase production in a single hypha could be monitored effectively using the new device. Until now, enzyme production in filamentous fungi has been tracked mainly by analyzing RNA or the secreted material level using enzymatic assays, electrophoresis or western blotting methods. Even those analysis methods using fusion proteins have used snapshots taken at specific times rather than analyzing growth and enzyme production in real time. This research offers a novel solution to the challenges in measuring fungal growth and morphology, enzyme production, and specific promoter strength, all of which have been difficult to measure simultaneously. The microfluidic device we have developed can measure these parameters simultaneously and in real time. We have also shown that subtle differences in polarity between cells and extracellular solutions can enable the detection of small amounts of enzymatic reactions. This finding applies to detecting enzymes corresponding to various substrates based on 4-MU and will contribute to studying enzyme secretion in other filamentous fungi.

The microfluidic device can also be used to study more complex fungal behavior, such as the branching and fusing different hyphae within a fungus, by branching and connecting channels in the middle. Furthermore, integrating multiple loading channels on a single chip allows the comparison of different fungal strains and the study of interactions between fungi with different genotypes. In addition to detection by imaging, techniques such as laser microdissection can be combined to extract sections of hypha from the hypha of interest accurately. Furthermore, the equipment and strategies presented here can be immediately extended to various other cell types, allowing a wide range of new studies to reveal the characteristics and mechanisms of long-term cell growth. Other new research areas for the future include improved layout for the microfluidic device, the integration of faster stage movement motors, automated image acquisition microscopy systems, optics and detectors with higher spatial and temporal resolution, and the development of a variety of fluorescent molecular markers.

## Data availability statement

The original contributions presented in the study are included in the article/Supplementary material, further inquiries can be directed to the corresponding authors.

## Author contributions

AI, YS, and WO helped in conceptualization and wrote the manuscript. AI performed the research. WO contributed to new reagents/analytic tools and funding acquisition. YS and WO supervised the study. All authors discussed the results and commented on the manuscript.

## Funding

This study is based on results obtained from a project, JPNP19001, commissioned by the New Energy and Industrial Technology Development Organization (NEDO). This study was also supported by Program on open innovation platform for industry academia co-creation (COI-NEXT) of Japan Science and Technology Agency (JST), Grant Number JPMJPF2211.

## Conflict of interest

The authors declare that the research was conducted in the absence of any commercial or financial relationships that could be construed as a potential conflict of interest.

## Publisher’s note

All claims expressed in this article are solely those of the authors and do not necessarily represent those of their affiliated organizations, or those of the publisher, the editors and the reviewers. Any product that may be evaluated in this article, or claim that may be made by its manufacturer, is not guaranteed or endorsed by the publisher.
