# Supplementary material for: A microfluidic device for simultaneous detection of enzyme secretion and elongation of a single hypha
Source: Front Microbiol. 2023 Mar 3;14:1125760. doi: 10.3389/fmicb.2023.1125760 (PMC10020217; doi:10.3389/fmicb.2023.1125760)
Supplement: Supplementary file 5 [file Data_Sheet_1.pdf]

	Primer name	Sequence (5' → 3')	Description
H2B-GFP expressing strains	*h2b* Fw	GGATCCTCTAGAGTCATGVATCCTCCAGAAGGTAAGGGTC	Histone *h2b* amplification
	h2b Rv	CATGCCTGCAGGTCATGCATCGAGCACCGTTGAAATAGCC	
	Inv *h2b*(*gfp*) Fw	TGAGCTGTACAAGTGAGAGGGGTTCATGACGACTGGGTTC	Inverse PCR for *gfp* transduction
	Inv *h2b*(*gfp*) Rv	GCCCTTGCTCACCATTTTCGTGGATGAGGAGTACTTGGTG	
	*gfp*_Fw	ATGGTGAGCAAGGGCGCC	*gfp* amplification
	*gfp*_Rv	TCACTTGTACAGCTCATCCATGCCG	
	Inv *pyr4* Rv	GGGAGCAGCGCTGATCCTGCATCCTGTGAGCCAAAC	Inverse PCR for *pyr4* transduction
	Inv p*yr4* Fw	GCAGTTGTCGACGATATACCCTGATTGTCGCTGGCG	
CBH1-GFP expressing strains	*cbh1* Fw	CAGTCCCGTGGAATTCTCACGGTGAATG	*cbh1* amplification
	cbh1 Rv	GAATTCCAGCCCTAGAAGCGCCAGGATCA	
	Inv *pyr4* Fw	AGCTCCGTGGCGAAAGCCTGACGCA	Inverse PCR for *pyr4* transduction
	Inv *pyr4* Rv	GATGCGCAGTCCGCGGTTGACTATTGGGT	
	*gfp*_Fw	ATGGTGAGCAAGGGCGCC	*gfp* amplification
	*gfp*_Rv	TCACTTGTACAGCTCATCCATGCCG	
	Inv *cbh1*(*gfp*) Fw	TCGGCCTGCACTCTCCAATCG	Inverse PCR for *gfp* transduction
	Inv *cbh1*(*gfp*) Rv	CTGAGCACGAGCTGTGGCCA	

Table S1. Primers used in this study.

Genomic DNA from QM9414 was used as a template to amplify the target gene. pUC118 vector was used for GFP-H2B expression and pU*h2b-gfp-pyr4* was created. The target gene was amplified in the same way for CBH1-GFP and pBluescriptII KS(+) and inserted into a vector to create pB*cbh1-gfp-pyr4*.

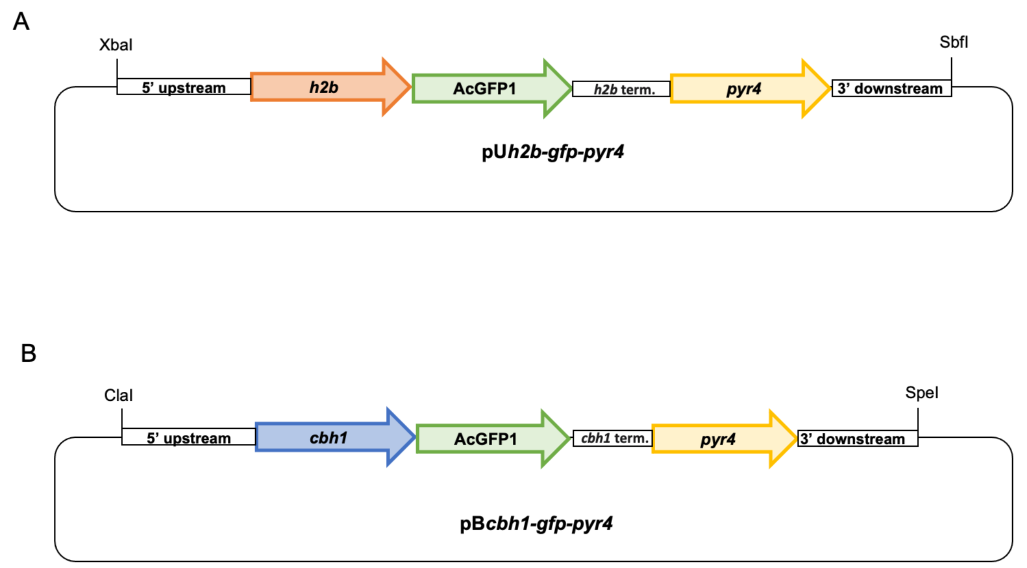

Fig. S1. Nuclear dynamics were observed with the aid of microdevices.

Plasmid map used in this study. (A) pU*h2b-gfp-pyr4*, (B) pB*cbh1-gfp-pyr4*.

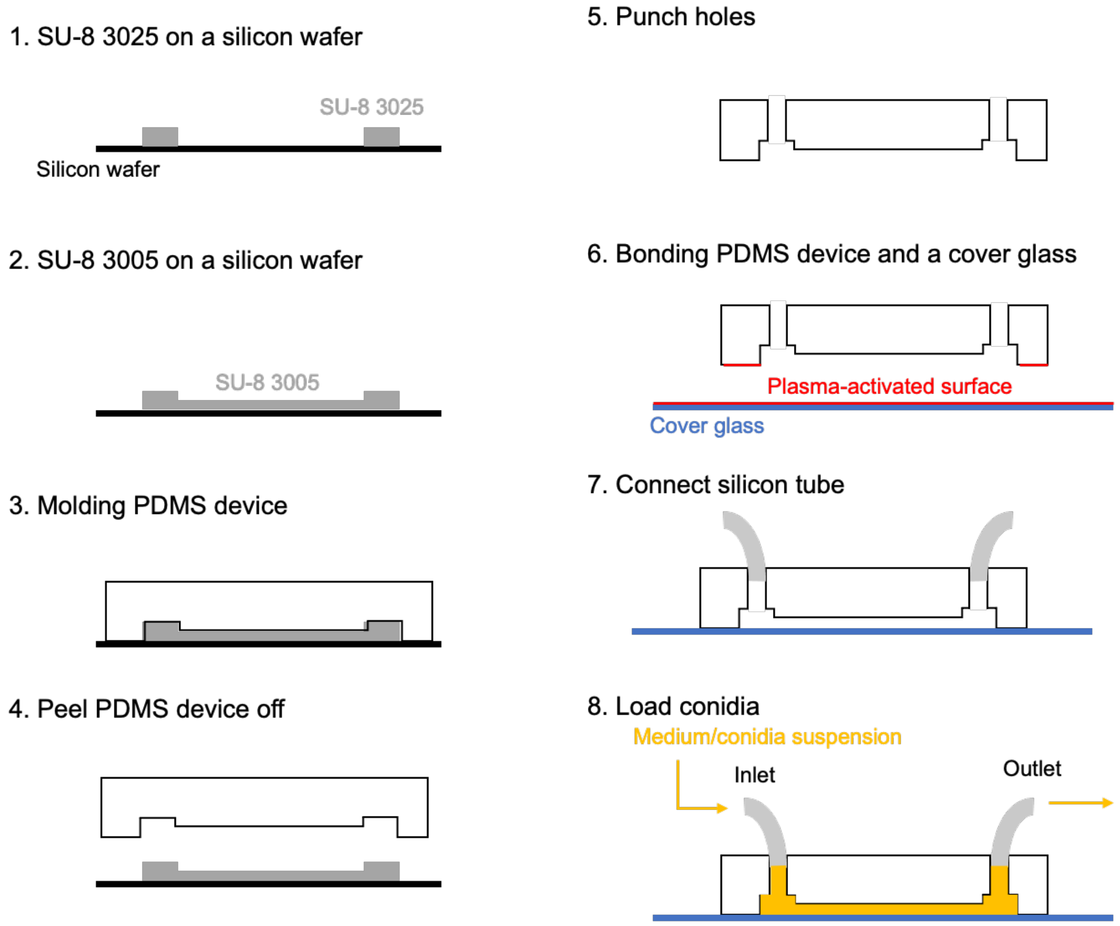

Fig. S2. The microfluidic device fabrication procedure.

More information on microfluidic device creation:

The CAD designs, created using ZunoRAPID software (Photron, Tokyo, Japan), were printed on photoresist-coated chrome-on-glass masks (CBL4006Du-AZP, Clean Surface Technology, Kanagawa, Japan) using a laser-drawing system (DDB-201-TW, Neoark, Osaka, Japan). The UV-exposed regions of the photoresist layer (AZP1350) were removed by NMD-3 (Tokyo Ohka Kogyo, Kawasaki, Kanagawa, Japan), and the exposed chromium was etched by MPM-E350 (DNP Fine Chemicals, Yokohama, Kanagawa, Japan). After removing the remaining photoresist layer using acetone (Wako, Osaka, Japan), the masks were rinsed with MilliQ water and air-dried.

An SU-8 mold for the PDMS device was made on a silicon wafer in 2 steps. First, to make observation channels, the wafer was coated with SU-8 3005 (Nippon Kayaku, Tokyo, Japan) using a spin-coater (MS-A150, Mikasa) at 500 rpm. for 10 s and then at 4,000 rpm for 30 s. After soft baking at 65°C for 1 min and 95°C for 10 min, the photoresist layer was exposed to UV using the mercury lamp of a mask-aligner (MA-20, Mikasa) at 22.4 mW/cm2 for 12 s. Post-exposure baking was performed at 65°C for 1 min and 95°C for 5 min, followed by exposure to SU-8 developer (Nippon Kayaku) and a 2-propanol

(FUJIFILM Wako Pure Chemical) rinse. The same procedure was repeated to fabricate trenches using SU-8 3025 (Nippon Kayaku) at 500 rpm for 10 s and then at 2,000 rpm for 30 s. Soft baking was performed at 65°C for 1 min and 95°C for 10 min, followed by UV expo-sure at 22.4 mW/cm^2 for 16 s and post-exposure baking at 65°C for 1 min and 95°C for 5 min.

The polydimethylsiloxane (PDMS) base and a curing agent (SYLGARD 184 Silicone Elastomer Kit, DOW SILICONES, Midland, MI, USA) were mixed at a ratio of 10:1, poured onto the SU-8 mold in a container, and de-gassed using a vacuum desiccator for 30 min. The PDMS was cured at 65˚C for 1 hour, and a 20 mm × 20 mm square PDMS pad was cut out using a blade. Two holes (Ø = 0.5 mm) in the PDMS pad were punched out for the inlet and outlet, cleaned briefly in ethanol with sonication, and then air-dried. The surface of the device and a coverslip (24 × 60 mm, thickness 0.12–0.17 mm, Matsumani, Bellingham, WA, USA) were activated using a plasma cleaner (PDC-32G, Harrick Plasma, Ithaca, NY, USA) and bonded together.

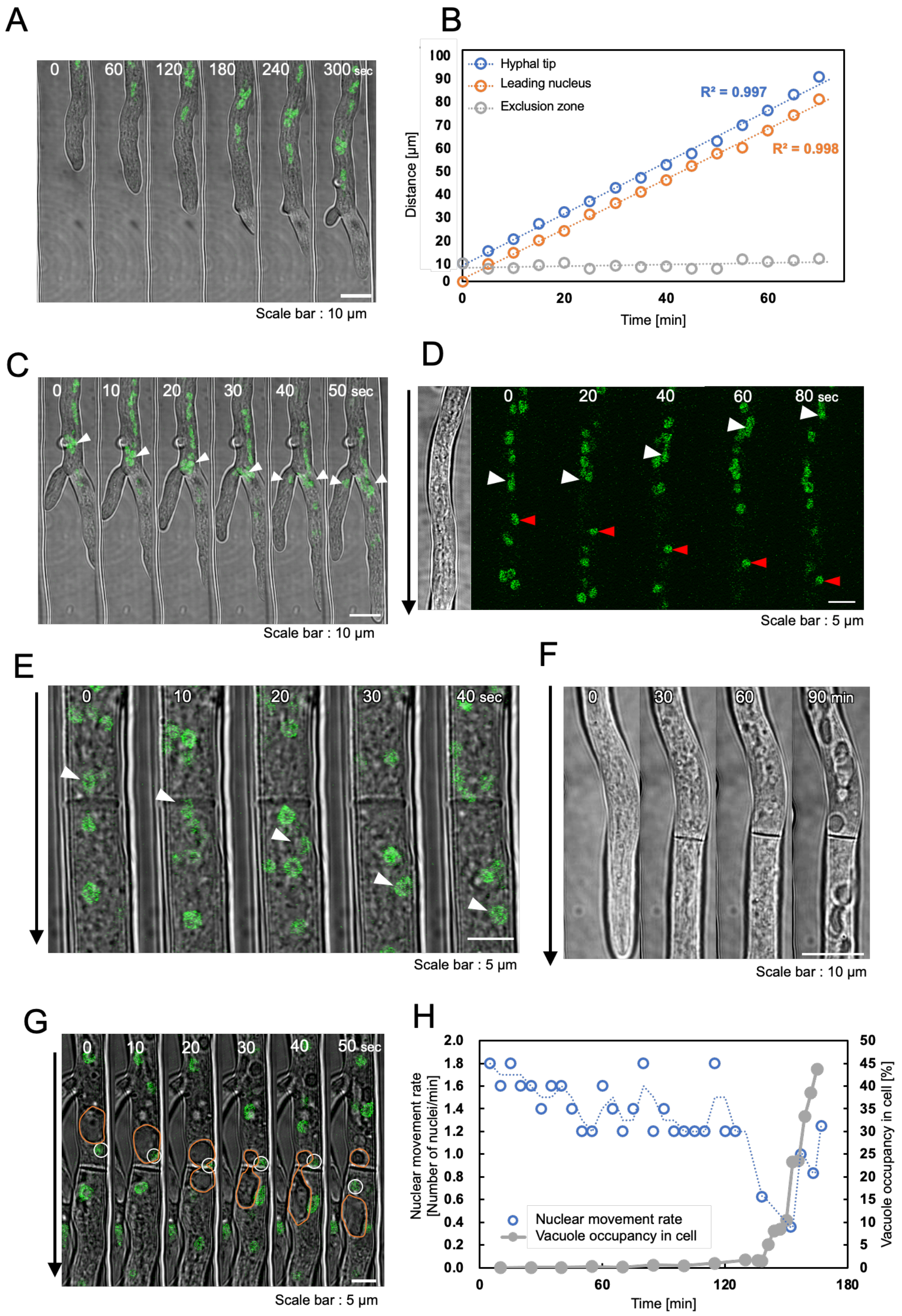

Fig. S3. Nuclear dynamics were observed with the aid of microdevices.

(A) Time-lapse imaging of long-range nuclear migration combined with bright-field and fluorescence microscopy. The extension of the hyphal tip, the migration of the leading nucleus, and the distance of the nuclear exclusion zone were observed. Scale bar: 25 μm. Channel width: 17.5 μm. The images are from Supplementary Movie S1. (B) Quantifying the extension of the hyphal tip, the migration of the leading

nucleus and the distance of the nuclear exclusion zone from the leading nucleus to the hyphal tip in (A). (C) Nuclear dynamics in hyphal branches. Scale bar: 10 µm. The images are from (A) and Supplementary Movie S1. Arrows indicate the direction of hyphal extension. (D) Retrograde nucleus. Scale bar: 5 µm. Channel width: 15 µm. Supplementary Movie S4. (advancing nucleus: red arrows, reverse direction: white arrows). (E) Nuclear dynamics at the septum. Scale bar: 10 µm. Channel width: 7.5 µm. The images are from Supplementary Movie S2. (F) As hypha ages, vacuoles enlarge. Scale bar: 10 µm. Channel width: 12.5 µm. (G) The nucleus was impeded in its passive movement by a large vacuole. Scale bar: 5 µm. Channel width: 15 µm. The image from Supplementary Movie S3. (vacuole: orange, nucleus: white). (H) Effect of vacuolar occupancy in hyphae on nuclear migration. Measurements were on the cell from (D).

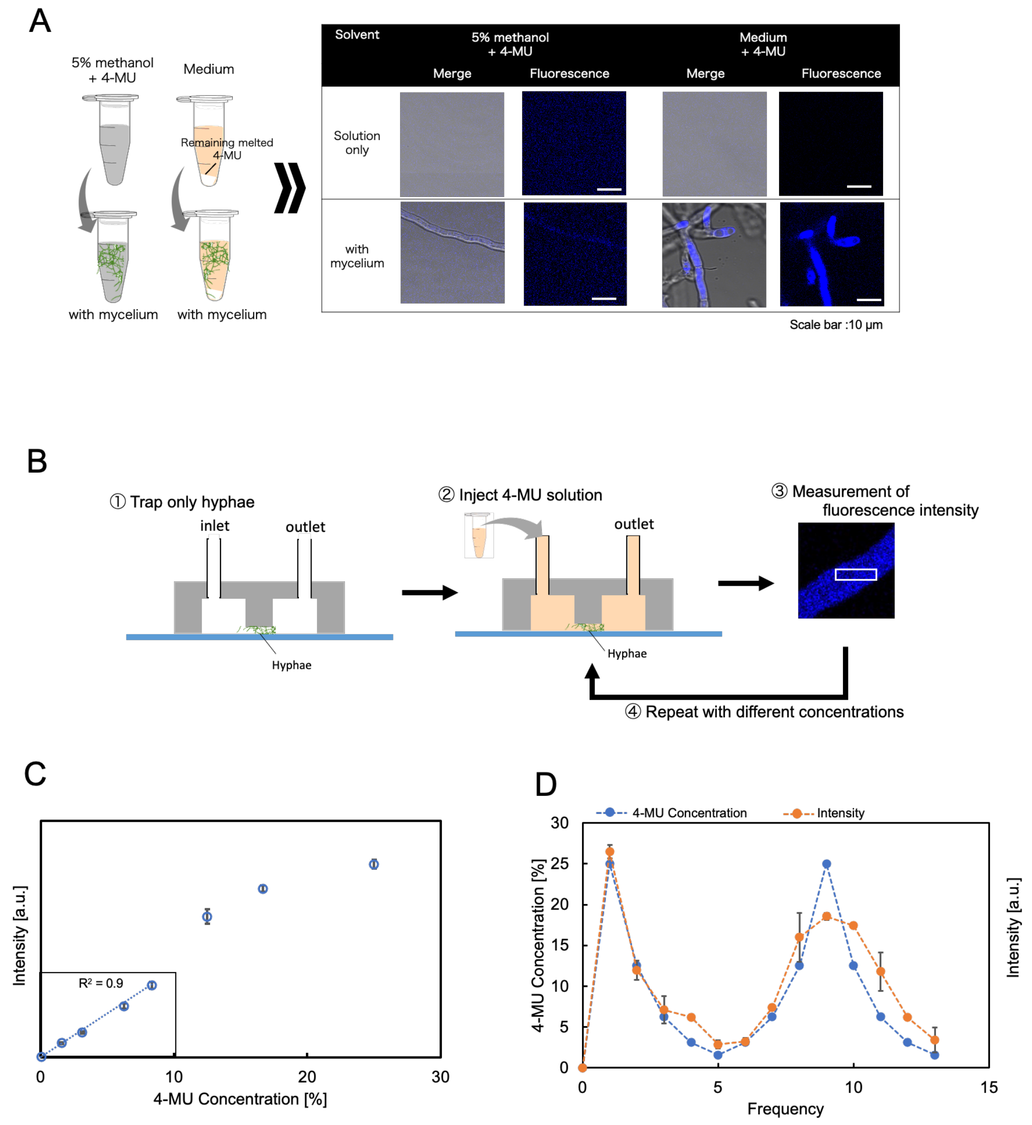

Fig. S4. Validation of cellulase detection assay with 4-MUC

(A) Differences in 4-MU fluorescence with and without methanol. When the fluorescent substance 4-MU was dissolved in 5% methanol, the solution fluoresced dimly, but the hyphae did not. When 4-MU was added to the medium, the solution did not fluoresce, but the hyphae fluoresced strongly. (B) Fluorescence intensity measurement method for (C) and (D). (C) Calibration curve of 4-MU and hyphal fluorescence intensity. At low concentration (R^2 = 0.9). (D) Intracellular levels of 4-MU fluctuate. 4-MU don't accumulate intracellularly and the fluorescence intensity is concentration-dependent.

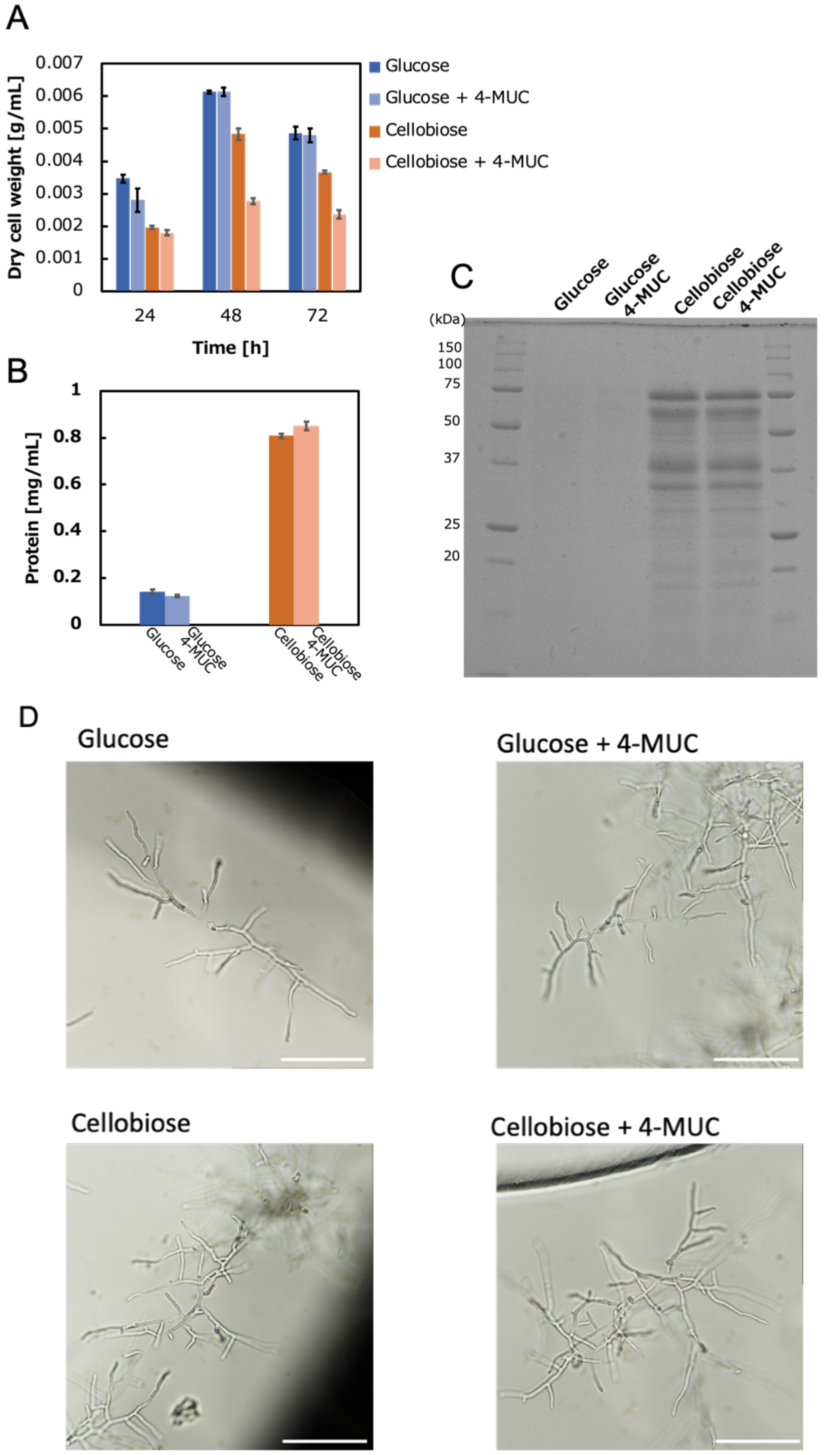

Fig. S5. The growth, morphology and enzyme production in the culture with 4-MUC.

Comparisons were made between 1% glucose medium with the addition of 1 mM 4-MUC and 1% cellobiose medium with the addition of 1 mM 4-MUC. (A) Dry cell weight transition. (B) Extracellular protein concentration at 48 hours of incubation. (C) SDS-PAGE of culture supernatant from (B). (D) Growth in 4-MUC-supplemented culture medium. Taken at 24 h of incubation. Scale bar: 100 µm.

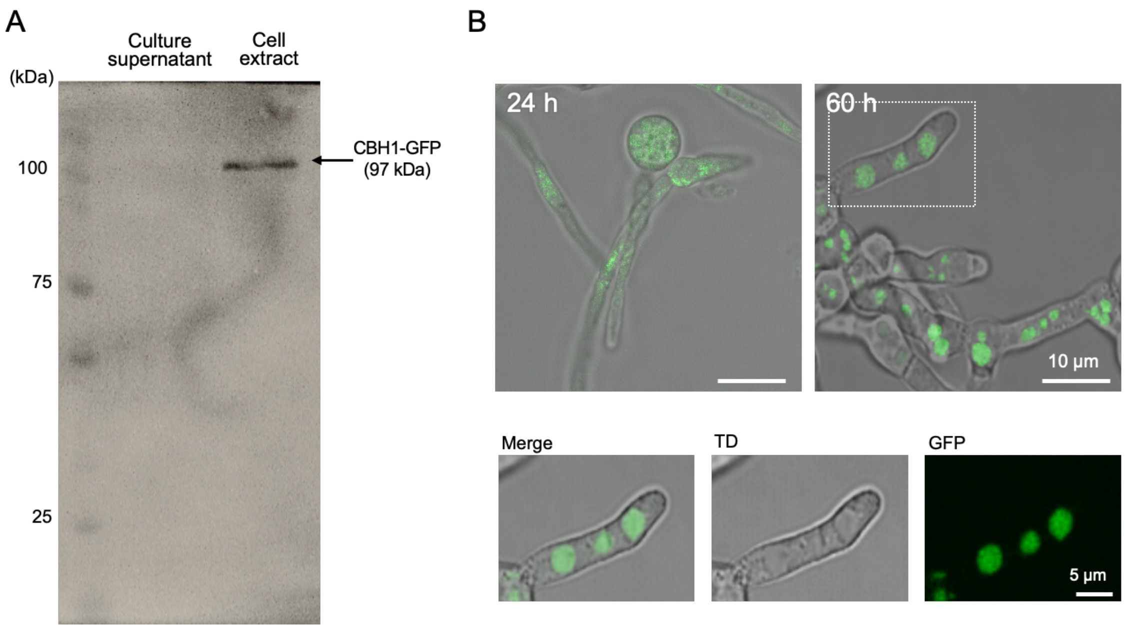

Fig. S6. Behavior of GFP in *T. reesei* PC*cbh1gfp*

(A) Western blot of culture supernatant and cell extracts. CBH1-GFP was not secreted. (B) Behavior of GFP in hyphae in flask culture; GFP accumulated in vacuoles at 60 h.

Movie legends

Movie S1

Time-lapse imaging of long-range nuclear migration with combined with bright-field and fluorescence microscopy at intervals of 30 second intervals. The extension of the hyphal tip, the migration of the leading nucleus, and the distance of the nuclear exclusion zone were observed. Scale bar: 25 μm.

Movie S2

Time-lapse imaging of long-range nuclear migration with combined with bright-field and fluorescence microscopy at intervals of 10 second intervals. Nuclear dynamics at the septum. Scale bar: 10 μm.

Movie S3

Time-lapse imaging of long-range nuclear migration with combined with bright-field and fluorescence microscopy at intervals of 10 second intervals. Nucleus was impeded in its passive movement by a large vacuole. Scale bar: 5 μm

Movie S4

Time-lapse imaging of long-range nuclear migration with combined with bright-field and fluorescence microscopy at intervals of 10 second intervals. Retrograde nucleus. Scale bar: 5 μm (reverse direction: white circle).
